# Therapeutic approaches to enhance natural killer cell cytotoxicity

**DOI:** 10.3389/fimmu.2024.1356666

**Published:** 2024-03-11

**Authors:** Terran D. Stenger, Jeffrey S. Miller

**Affiliations:** Division of Hematology, Oncology, and Transplantation, Department of Medicine, Masonic Cancer Center, University of Minnesota, Minneapolis, MN, United States

**Keywords:** natural killer cell (NK cell), NK cell, cytotoxicity, NK cytotoxicity, immunotherapy, TriKEs, cancer immunotherapy, NK cell engager

## Abstract

Enhancing the cytotoxicity of natural killer (NK) cells has emerged as a promising strategy in cancer immunotherapy, due to their pivotal role in immune surveillance and tumor clearance. This literature review provides a comprehensive overview of therapeutic approaches designed to augment NK cell cytotoxicity. We analyze a wide range of strategies, including cytokine-based treatment, monoclonal antibodies, and NK cell engagers, and discuss criteria that must be considered when selecting an NK cell product to combine with these strategies. Furthermore, we discuss the challenges and limitations associated with each therapeutic strategy, as well as the potential for combination therapies to maximize NK cell cytotoxicity while minimizing adverse effects. By exploring the wealth of research on this topic, this literature review aims to provide a comprehensive resource for researchers and clinicians seeking to develop and implement novel therapeutic strategies that harness the full potential of NK cells in the fight against cancer. Enhancing NK cell cytotoxicity holds great promise in the evolving landscape of immunotherapy, and this review serves as a roadmap for understanding the current state of the field and the future directions in NK cell-based therapies.

## 1 Introduction

While improvements in cancer prevention and treatment have led to a decrease in cancer deaths per capita, it remains the second leading cause of death worldwide, highlighting the need for novel treatment strategies (1). Following several observations that tumors are capable of inhibiting and evading the immune system, immunotherapy has come to the forefront of cancer research and drug development following the turn of the twenty-first century.

Some cancers, for example, have been shown to upregulate inhibitory ligands for immune cells, like programmed cell death ligand 1 (PD-L1). Upon binding to PD-1 on T and other immune cells, strong inhibition of cytotoxic activity is observed, and a similar inhibitory interaction was discovered through cytotoxic T-lymphocyte antigen-4 (CTLA-4) (2). In response, the researchers responsible for identifying these immunosuppressive interactions sought to block receptor-ligand binding and restore optimal cytotoxic activity against cancer cells. This was achieved through the use of monoclonal antibodies, leading to the development of therapeutics that significantly improved the likelihood of survival across several types of cancer. In metastatic malignant melanoma, for example, the long-term survival rate increased from 5% to a staggering 50% upon combined anti-CTLA-4 and anti-PD-1 monoclonal antibody treatment (3). The 2018 Nobel Prize in Physiology or Medicine was awarded for these discoveries inhibiting negative regulation of the immune system in cancer, more than a quarter of a century since it was last given for discoveries in the field of cancer therapy. While early research in immunotherapy, like the pioneering studies described above, tends to focus on CD8+ T cells, natural killer cells have recently become attractive candidates for immunotherapy, in part for their positive safety profile. Compared to chimeric antigen receptor (CAR) T cell therapy, for example, which is not appropriate for all patients due to significant side effects including cytokine release syndrome and neurotoxicity, NK cell therapy has been shown to be more tolerable and can be generated for off-the-shelf usage.

## 2 NK cell biology

Natural killer cells are vital components of the innate immune system, with potent abilities to recognize and eliminate virus-infected and/or cancer cells (4–6). Unlike CD8+ T cells and other adaptive immune cell types, NK cell receptors are germline-encoded and do not undergo rearrangement, nor do they display specificity (7). Instead, they display several activating and inhibitory receptors (see **Table 1**) and the net signal through these receptors determines whether or not the NK cell will become activated, kill target cells, and produce cytokines. As a result of the relatively low specificity of these receptors, NK cells do not require prior sensitization to exert their cytotoxic effects, nor are they major histocompatibility complex (MHC) restricted (8). NK cell-based therapies, therefore, do not require the time-intensive process of scaling up autologous cells, but rather can undergo haploidentical and allogeneic adoptive transfer without high risk of graft versus host disease (9, 10).

**Table 1:** Activating and inhibitory receptors found on NK cells.

Receptor Family	Receptor	Function	Ligand(s)
Fragment Crystallizable Receptor (FcR)	CD16	Activating	IgG
Natural Cytotoxicity Receptor	NKp30	Activating	Bacterial, viral, and tumor-associated ligands
	NKp44		
	NKp46		
C-type lectin	NKG2A	Inhibitory	HLA-E
	NKG2C	Activating	HLA-E
	NKG2D	Activating	MICA MICB ULBP1-ULBP6 Rae-1
	NKG2E	Activating	HLA-E
Killer-Cell Immunoglobulin-Like receptor (KIR)	2DS1-2DS5, 3DS1	Activating	HLA Class I or unknown
	2DL1-2DL3, 2DL5, 3DL1-3DL3	Inhibitory	
	2DL4	Activating and Inhibitory	

NK cells, like CD8+ T cells, arise from the lymphoid lineage of hematopoietic stem cells best known for their cytotoxic ability, and for their production of inflammatory cytokines upon activation, like IFN-γ and TNF-α, as well as CC-chemokines (11). Also akin to CD8+ T cells, NK cells directly kill target cells through degranulation of perforin- and granzyme-loaded vesicles and Fas ligand-dependent cytotoxicity. However, unlike T cells, these lymphocytes do not require prior sensitization or specific antigen recognition to mount their cytotoxic response, and instead possess activating and inhibitory innate receptors capable of distinguishing between healthy cells and cells altered by infection or malignancy (12). CD16, for example, binds the Fc-portion of cell surface-bound IgG antibodies, leading to formation of an immunological synapse and degranulation. This ultimately results in lysis of the target cell, a process termed antibody-dependent cell-mediated cytotoxicity (ADCC) (13). Through several receptors, including members of the natural cytotoxicity family (NKp30, NKp44, NKp46) and C-type lectin family (NKG2C, NKG2D, NKG2E), NK cells are activated upon detection of transformed cells (the “induced-self hypothesis). Another member of the C-type lectin family, NKG2A, along with inhibitory members of the killer cell inhibitory receptor (KIR) family, inhibits NK function upon binding to class I HLA ligands, making NK-mediated cytolysis more likely after downregulation of HLA (the “missing-self hypothesis). In part due to the lack of antigen-specificity of these receptors, NK cells are not generally considered mediators of graft-versus-host disease (GvHD) and have even been shown to suppress it, highlighting their potential for accessible and standardized allogeneic administration (14). Additionally, NK cells have been shown to infiltrate several tumor types, and are positively correlated with patient outcomes (15).

For these reasons, and because of their crucial role in tumor immunosurveillance and anti-tumor responses, NK cells are attractive candidates for immunotherapy. In recent years, there has been a growing interest in therapeutic approaches to enhance NK cell cytotoxicity for such purposes despite immunosuppression within the tumor microenvironment. These strategies aim to intensify NK cell function, improving their ability to recognize and eliminate tumor cells by leveraging unique properties of NK cells, such as their natural cytotoxicity and their diversity of activating and inhibitory receptors.

## 3 Immunosuppression in the tumor microenvironment

As with most immune responses, NK cell activity is inhibited by the immunosuppressive tumor microenvironment, as a result of several physical, molecular, and cellular barriers. As tumors rapidly proliferate and expand, the demand for oxygen becomes higher than the available supply (16). This, along with the high metabolic activity and suboptimal vasculature within solid tumors, leads to hypoxia. Such conditions lead to significant changes in the transcriptome of NK cells, including downregulation of proinflammatory cytokines and chemokines like IFNγ, TNFα, GM-CSF, CCL3, and CCL5 (17). This is likely due, in part, to the restricted metabolic capacity of NK cells in hypoxic conditions (18).

Immunosuppressive cells like regulatory T cells (Tregs) and myeloid-derived suppressor cells (MDSCs) are found in high concentrations within solid tumors, producing several cytokines that exert inhibitory effects on NK cells (19). For example, TGFβ, released by Tregs, MDSCs, and other stromal cells, leads to the downregulation of activating receptors of NK cells, including NKG2D and NKp30 (20). TGFβ signaling also leads to impaired ADCC and IFN-γ production by activating the transcription factor SMAD3 (21). Other immunosuppressive molecules, such as IL-10 and prostaglandin E2 lead to similar defects in the anti-tumor capabilities of NK cells. Some tumors have also been shown to evade NK responses by upregulation of molecules that push NK cells toward a more tolerant state, such as the NKG2A ligand HLA-E (20). Such findings highlight the importance of developing novel therapeutics that overcome the immunosuppressive tumor microenvironment and enhance NK cytotoxicity.

## 4 Approaches to enhance NK cytotoxicity

One promising approach to enhance the cytotoxic effects of NK cells therapeutically is through the use of cytokines. Interleukin-2 (IL-2) has been extensively studied for its role in enhancing NK cell cytotoxicity. IL-2 acts as a growth factor for NK cells, promoting their proliferation and activation (22). It also enhances NK cell-mediated cytotoxicity through both the upregulation of activating receptors and the production of cytotoxic molecules (23, 24). Another cytokine, interleukin-15 (IL-15), has gained attention for its critical role in NK cell development, survival, and function (25–28). The IL-15 and IL-2 receptor on NK and other immune cells consists of identical beta (CD122) and gamma chains (common gamma chain [γc] or CD132), with differences in the alpha chain differentiating the two cytokines’ functions (29–31). IL-15 administration, like IL-2, has been shown to stimulate NK cell cytotoxicity and promote anti-tumor responses, though with less toxicity and absence of Treg stimulation, which present challenges to IL-2-based therapies (32–35). Additionally, cytokines such as interleukin-12 (IL-12), interleukin-18 (IL-18), and interleukin-21 (IL-21) have demonstrated the ability to modulate NK cell activity and enhance their antitumor function through various, often interlaced, mechanisms (36–39).

Monoclonal antibodies (mAbs) have also emerged as powerful tools in cancer immunotherapy, especially in regard to their ability to activate NK cells. One notable class, NK checkpoint blockade antibodies, block inhibitory receptors on NK cells or inhibitory ligands on tumor cells, thereby unleashing NK cell cytotoxicity. For example, several checkpoint blockade antibodies originally found to act upon T cells have shown to also improve NK cell cytotoxicity. For example, mAbs targeting the programmed death receptor axis PD-1/PD-L1, T cell immunoglobulin and ITIM domain (TIGIT), lymphocyte activation gene-3 (LAG-3), and T cell immunoglobulin mucin-3 (TIM-3) are known to enhance NK cell function and improve anti-tumor responses (40–43). Another recent example is monalizumab, which targets NKG2A, an inhibitory receptor on NK cells. By blocking NKG2A and inhibiting its binding with HLA-E, monalizumab enhances NK cell cytotoxicity against tumor cells (44). Furthermore, mAbs targeting pan-cancer antigens, such as rituximab (targeting CD20), trastuzumab (targeting HER2), and cetuximab (targeting EGFR), have been shown to rely on NK cell-mediated ADCC for their anti-tumor effects, though other immune cells including macrophages, dendritic cells, and granulocytes are also known to contribute (45–48). NK cells, through the recognition of antibody-coated target cells by CD16, a potent Fcγ-receptor, exert their cytotoxic activity and are major contributors to the efficacy of these mAbs (7). However, surface CD16 can be rapidly shed by A Disintegrin And Metalloproteinase 17 (ADAM17), leading to inhibition of NK cell responses. ADAM17 inhibitors have been developed, but this remains a major issue in the therapeutic efficacy of NK cell-based therapies (49).

In addition to cytokines and monoclonal antibodies, NK cell engagers have emerged as a promising strategy to enhance NK cell cytotoxicity. Such molecules are designed to bridge NK cells with target cells, thereby directing NK cell-mediated cytotoxicity towards specific tumor cells. These engagers can be in the form of bispecific antibodies or engineered proteins that simultaneously bind to NK cell activating receptors and tumor antigens. For example, Trispecific Killer Engagers (TriKEs) have been developed to target CD16 on NK cells, target a tumor antigen, and deliver IL-15 (50). By binding to CD16, TriKEs activate NK cells, while the tumor antigen-specific component directs NK cell cytotoxicity towards tumor cells. Additionally, the inclusion of IL-15 provides sustained stimulation and proliferation of NK cells. Other NK cell engagers, such as TriNKET, ROCK, and ANKET, have also been explored to enhance NK cell-mediated cytotoxicity against tumor cells (51–54).

In this review, we aim to provide a comprehensive overview of the current state of therapeutic approaches to enhance NK cell cytotoxicity in an anti-tumor setting. We will explore the role of cytokines, monoclonal antibodies, and engagers, in augmenting NK cell function, highlighting recent clinical trials for each approach (summarized in **Table 2**). To conclude, we will highlight important characteristics that should be considered when selecting an NK cell product to combine with these therapeutic approaches. Furthermore, we will discuss different schools of thought, controversies, fundamental concepts, current research gaps, and potential developments in each area. By presenting a balanced perspective, we hope to shed light on the recent advancements and future prospects of these therapeutic strategies.

**Table 2:** Selected clinical trials aiming to enhance NK cytotoxicity.

Agent of Interest	Treatment Approach	Malignancy/patient profile	Trial Phase (Status)	ClinicalTrials.gov Identifier (Trial Name)
Cytokines				
IL-2	In combination with haploidentical NK cell adoptive transfer post-chemo and/or radiotherapy	Pediatric, adolescent, and young adult refractory or metastatic sarcomas	Phase I/II (recruiting)	NCT05952310 (SANKOMA)
Saltikva (*Salmonella typhimurium* expressing IL-2)	Oral administration while on chemotherapy	Stage IV metastatic pancreatic cancer	Phase II (recruiting)	NCT04589234
MDNA11 (“beta-only” IL-2 – human albumin fusion protein)	Monotherapy, or in combination with pembrolizumab	Advanced or metastatic solid tumors	Phase I/II (recruiting)	NCT05086692 (ABILITY-1)
N-803	Monotherapy, or in combination with BCG	BCG unresponsive high grade non-muscle invasive bladder cancer	Phase II/III (recruiting)	NCT03022825 (QUILT-3.032)
N-803	In combination with standard-of-care chemotherapy, aldoxorubicin HCl, and PD-L1 t-haNK cells	Locally advanced or metastatic pancreatic cancer	Phase II (recruiting)	NCT04390399 (QUILT-88)
Monoclonal Antibodies				
Rituximab	In combination with allogeneic NK cells, high-dose chemotherapy, and stem cell transplant	Recurrent or refractory B cell non-Hodgkin’s lymphoma	Phase II (completed)	NCT03019640
Daratumumab	In combination with BCMA-targeted CAR NK cells	Relapsed or refractory multiple myeloma	Phase I (recruiting)	NCT05182073
Mogamulizumab	In combination with third-party NK cells	Relapsed or refractory cutaneous T cell lymphoma or adult T cell leukemia/lymphoma	Phase I (recruiting)	NCT04848064
Monalizumab and trastuzumab	Combination therapy	Metastatic or locally incurable HER2+ breast cancer	Phase II (active, not recruiting)	NCT04307329
Monalizumab and durvalumab	Combination therapy	Locally advanced, unresectable non-small cell lung cancer	Phase III (recruiting)	NCT05221840 (PACIFIC-9)
Engagers				
GTB-3550 (anti-CD33 TriKE)	Monotherapy	High risk CD33+ hematological malignancies	Phase I/II (terminated)	NCT03214666
IPH6501 (anti-CD20 ANKET)	Monotherapy	Relapsed or refractory B cell non-Hodgkin lymphoma	Phase I/II (not yet recruiting)	NCT06088654
DF1001 (anti-HER2 TriNKET)	In combination with nivolumab or nab-paclitaxel	Advanced HER2+ solid tumors	Phase I/II (recruiting)	NCT04143711
AFM13 (anti-CD30 ROCK)	Monotherapy	Relapsed or refractory CD30+ T cell lymphoma or transformed mycosis fungoides	Phase II (active, not recruiting)	NCT04101331 (REDIRECT)

## 5 Cytokines

### 5.1 IL-2

Despite their ability to lyse target cells without prior sensitization, NK cell effector function can be enhanced by exposure to inflammatory cytokines, first demonstrated in the context of IL-2. Currently, 75 clinical trials are underway that involve the use of IL-2 (www.clinicaltrials.gov). Discovered in 1976, IL-2, like other members of the common gamma-chain family, is a proinflammatory cytokine known to stimulate lymphocyte populations (55, 56). In the late 1970s, it was observed that transplant patients receiving immunosuppressive drugs developed high rates of cancer, leading to the hypothesis that suppressed activity of lymphocytes, including NK cells, must contribute to the development of cancer (57, 58). In the early 1980s, following seminal studies in NK biology, this hypothesis was confirmed when it was found that patients with cancer exhibit decreased peripheral-NK cell function, including ADCC and natural cytotoxicity (59, 60). At the same time, groups studying the effects of IL-2 on T cells found that it also increased cytotoxicity in NK cells *in vitro* (61). Such reports provided the basis for a study showing that by culturing dysfunctional NK cells from cancer patients with IL-2, cytotoxic activity was not only restored, but elevated compared to levels seen in healthy controls (62).

Based on this important finding, researchers began a clinical trial testing the administration of autologous, activated NK cells in combination with IL-2 in various metastatic cancers (63, 64). While IL-2 alone or activated NK cells alone showed little tumor regression, the combined treatment resulted in marked regression in several patients, leading to its approval by the FDA (64, 65). It was approved for metastatic renal cell carcinoma (RCC) in 1992 and for metastatic melanoma in 1998 and is still in use today for both cancers.

IL-2 is also being tested for clinical use in other tumor settings, mainly in combination with other forms of treatment. At least ten clinical trials are currently underway involving IL-2 in combination with tumor-infiltrating lymphocytes (TILs). While variations exist between each trial, the general strategy involves the expansion of TILs from small, surgically resected tumor samples and, following lymphodepletion, are readministered into the patient, followed by IL-2 treatment (66). Cancer types currently being tested include advanced cervical (NCT05475847), lymphocytic leukemia (NCT04155710), relapsed or metastatic melanoma (NCT05903937, NCT05628883, NCT04812470), and head and neck cancers (NCT03991741). There has also been a recent interest in combining IL-2 with other forms of immunotherapy, such as in combination with immune checkpoint blockade (NCT04562129, NCT04165967, NCT05493566), or in combination with the Bacillus Calmette-Guerin (BCG) vaccine (NCT03928275).

Several additional trials combine IL-2 treatment with adoptive transfer of activated NK cells. One phase I/II study combines this with localized irradiation (NCT05952310). 24-48 hours after myeloablative chemotherapy and irradiation, patients receive one of two haploidentical NK cell infusions, with the other infusion given 4 days later. Simultaneously, IL-2 is administered every 48 hours subcutaneously for six total doses (results not yet available). In 2021, another clinical trial utilizing adoptive transfer of NK cells with IL-2 administration received fast track designation by the FDA (67). The study utilized hematopoietic stem cell-derived NK cells from CD34+ human placental cells and culturing them in the presence of several cytokines, including IL-7, IL-15, and IL-2 (NCT04310592). *In vivo*, such cells were shown to have increased expression of activating receptors NKG2D, NKp46, and NKp44, as well as increased antitumor cytotoxicity, compared to peripheral blood-derived NK cells (68). Following deletion of CBLB, a negative regulator of lymphocyte activity, proliferative and effector function was further increased. The cell therapy, CYNK-001, was tested in the setting of acute myeloid leukemia (AML) in combination with IL-2 administration. Early results show low response rates and indicate that IL-2 administration stimulated Treg populations without enhancing NK engraftment, and the study is no longer accepting enrollment (69). Other trials that also utilize the combination of IL-2 and NK cells, as well as the administration of mAb to the treatment regimen, will be discussed in future sections.

Despite the historical success of IL-2-based treatments, two major difficulties have since been encountered. First, it was observed early-on that high dose IL-2 administration, which is often required due to its short half-life, can lead to severe toxicity, thereby limiting treatment duration and interfering with patient safety (70, 71). More common side effects include rash, hypotension, thrombocytopenia, neurologic symptoms, and gastrointestinal symptoms (72–75). However, life-threatening capillary leak syndrome, leading to pulmonary and cerebral edema, respiratory distress, and heart failure can develop following intravenous administration of high dose IL-2. As IL-2 interacts with several aspects of the immune system, widespread pro-inflammatory cytokine release and lymphocyte activation is observed, leading to increased capillary permeability and eventual organ damage (76). To overcome this challenge, alternative methods of delivery are being explored clinically through the use of IL-2 expression in attenuated Salmonella (NCT04589234) and oncolytic adenoviruses (77). Such viruses are only capable of propagation within cancerous cells, leading to lysis and increased immune infiltration (78). TILT-123, a recently developed oncolytic adenovirus, contains an IL-2 as well as a TNFα coding region, to further elevate the immune response (79). Preclinical data showed promising results, and the treatment is now undergoing clinical assessment in ovarian (NCT05271318), advanced melanoma (NCT04217473), and other solid tumor settings (NCT04695327) (80).

Another issue observed with IL-2 administration is T regulatory cell (Treg) development and subsequent immune suppression. One study found that patients with metastatic cancers that showed no response to IL-2 treatment had significantly higher ICOS+ Tregs, an especially proliferative and immunosuppressive subset, than patients who responded to treatment (81). This suggests a direct role of Treg stimulation limiting the effectiveness of IL-2-based therapies. A recent approach to combat such suppression is through the generation of IL-2 mutants that exhibit selective binding to NK cells. The IL-2 receptor (IL-2R) exists in three forms, low-affinity (IL2Rα or CD25), intermediate-affinity (IL2Rβ or CD122 and cγ or CD132), and high-affinity (IL2Rα, IL2Rβ, and cγ) (82). NK cells, unlike Tregs, typically express the intermediate-affinity receptor, prompting the generation of a “beta-only” IL-2 (83). Known as MDNA11, it exists as a fusion to human albumin that was shown to improve pharmacokinetics. In preclinical models, it was shown to selectively bind IL-2Rβ, and therefore had significantly reduced Treg stimulation compared to IL-2. Additionally, the improved pharmacokinetics resulted in durable responses in animal studies with only once weekly dosing. Currently, MDNA11 is being tested in a phase I/II clinical trial as a monotherapy and in combination with immune checkpoint blockade (NCT05086692).

The historical success of IL-2-based therapies has been instrumental in advancing cancer treatment. In part through restoring and elevating NK cell cytotoxicity, IL-2 treatment has led to improved patient outcomes in conditions such as metastatic renal cell carcinoma and metastatic melanoma. These early studies have aided in the development of numerous ongoing clinical trials testing novel combinations and delivery methods of IL-2 to enhance NK cell cytotoxicity. Another vital cytokine, IL-15 plays a crucial role in the stimulation of lymphocyte populations, and its potential to boost NK cell function presents new opportunities for cancer therapy.

### 5.2 IL-15

IL-15, like IL-2, is a pro-inflammatory member of the common gamma-chain cytokines (84). It also signals through similar receptor components as IL-2, but it is trans-presented at high affinity with IL-15 receptor-alpha (IL-15Rα) (85). It also shows less systemic toxicity than IL-2 and does not lead to stimulation of Tregs (86–88). Though IL-15 was originally discovered as an inducer of T cell proliferation, its effect on NK cells was later discovered (29). For example, it was found that IL-15 and IL-15Rα deficient mice lack peripheral NK cell populations (26). It was also discovered that IL-15 enhances the survival, proliferation, and cytotoxicity of NK cells, resulting in improved anti-tumor capabilities (89, 90).

Although IL-15 is not currently approved in any form, the FDA will soon consider approval for an IL-15 in combination with BCG for non-muscle invasive bladder cancer (NMIBC). In 2009, targeted mutagenesis of human IL-15 led to the development of an IL-15 superagonist with 4-5-fold increases in effector cell proliferation and target cell lysis (91). The pharmacokinetics and functionality of the superagonist was further improved when complexed with an IL-15Rα-Fc fusion protein, resulting in significantly enhanced NK cell responses *in vivo* (92, 93). This therapeutic (N-803), in combination with BCG, was found to reduce tumor burden in a NMIBC model in rats (94). Specifically, the antitumor response was dependent on increases in the infiltration, proliferation, and activation of NK cells. Based on these findings, a Phase Ib clinical trial was started in 2014 to test BCG plus N-803 in human NMIBC patients who had not previously received BCG (NCT02138734). The trial enrolled nine patients, and, remarkably, complete response (CR) was achieved in all nine patients, and they all remained disease free six years post treatment (95). Simultaneously, the same treatment was tested in a Phase II/III trial with BCG-unresponsive patients, an especially difficult-to-treat form of NMIBC (NCT03022825). Findings from this trial revealed a 71% CR rate, with a median duration of response of 26.6 months (96). These observations provided the basis for the FDA to accept a Biologics License Application for N-803 plus BCG in BCG-unresponsive NMIBC. However, in May of 2023 the FDA’s decision to approve it was postponed because of deficiencies in data on duration of response and safety profiles, though it seems likely that FDA approval will eventually be reached (97, 98).

N-803 is also being tested in combination with PD-L1 targeting high-affinity NK (t-haNK) cells. t-haNKs are NK92 cells engineered to express both a PD-L1-targeting CAR and high-affinity CD16. PD-L1 t-haNKs were shown to retain expression of native NK receptors and inhibit growth of several tumor types *in vivo* (99, 100). A phase II clinical trial is testing these cells in combination with N-803 and standard-of-care chemotherapy in patients with locally advanced or metastatic pancreatic cancer (NCT04390399). Additionally, a phase I trial will test CD19 t-haNKs in combination with N-803 and rituximab in patients with relapsed or refractory non-Hodgkin Lymphoma (NHL) (NCT05618925). Other clinical trials involve N-803 in combination with various other forms of immunotherapy, such as cancer vaccines and pembrolizumab (NCT05642195, NCT05445882, NCT05096663).

IL-15 in combination with other NK-based therapies are also being tested. For example, two ongoing phase I clinical trials are treating hematologic malignancies with CAR NK cells and membrane-bound IL-15 (mbIL-15), which has been shown to increase NK cell proliferation and cytotoxic function in preclinical studies in an autocrine fashion (NCT04623944, NCT05020678) (101). Haploidentical, IL-15-stimulated NK cells are also being tested in the setting of hematologic malignancies post-hematopoietic stem cell transplant. Prior clinical trials with these cells in refractory cancers showed feasibility and safety, with some clinical benefit (102, 103).

Recently, continuous IL-15 treatment has been shown to lead to NK cell exhaustion consisting of decreased proliferation, antitumor function, and viability *in vitro* and *in vivo* (104). However, this was combated by both mTOR inhibition and treatment schedules which include gaps in IL-15 administration. Additionally, while toxicity related to IL-15 is less likely than with IL-2, some patients still develop toxicity such as weight loss, rash, and hypotension (though capillary leak syndrome has not been observed) (105). Similar to IL-2, alternative methods of administration (such as through oncolytic viruses) may alleviate these IL-15 toxicities (88). Such consequences of IL-15 dosing strategies, both in terms of toxicity and IL-15-mediated exhaustion, should be considered in clinical trial design.

IL-15, like IL-2, holds great potential for boosting NK cell function and enhancing the immune response against cancer. This is exemplified by the ongoing trials, particularly in NMIBC, where the combination of IL-15 with BCG has shown remarkable success, achieving complete responses in patients, and raising the prospect of FDA approval. Furthermore, IL-15’s role in combination with other NK-based therapies, such as CAR NK cells and membrane-bound IL-15 (mbIL-15), presents an exciting frontier in the treatment of hematologic malignancies.

## 6 Monoclonal antibodies

### 6.1 Tumor-specific mAb

Antigens differentially expressed on malignant cells with minimal expression on healthy tissue, known as pan-cancer antigens, represent optimal targets for immunotherapy, including mAb treatment (106). Targeting antigens with such expression patterns, like CD20, epidermal growth factor receptor (EGFR), human epidermal growth factor receptor 2 (HER2), and GD2 disialoganglioside results in limited off-target effects and manageable toxicity (107). Following mAb administration, NK-mediated ADCC is considered to be the major mechanism of action as, unlike other Fc-receptor expressing cell types, NK cells lack inhibitory Fc-receptors (108). Such synergism leads to elevated immune responses, and the combination of NK cells with tumor-targeting monoclonal antibodies is being tested in several clinical trials.

A monoclonal antibody targeting CD20, which is expressed on B cells and upregulated in hematological malignancies, was the first FDA approved mAb for the treatment of cancer (109). While the exact mechanism and ligand of CD20 in B cell biology remains unknown, animal models and studies in humans with mutated MS4A1, the gene encoding it, suggest it is required for efficient B cell receptor signalling (110). Aside from B cells, CD20 expression is limited in healthy tissue. While discrepancies exist between patients and cancer type, CD20 expression is generally high in B cell malignancies, highlighting its potential for targeting with mAbs (111). The first anti-CD20 mAb, rituximab, is a chimeric mouse/human mAb and was originally thought to cause target cell lysis through binding of C1q and subsequent complement activation as well as through transmission of apoptotic signals (112, 113). However, it is now known that FcR mechanisms are likely the dominant mediator of clinical success (114).

Rituximab became the first FDA approved therapeutic antibody in 1997 for various forms of NHL, and since then several new CD20 mAbs, alone and in combination with other drugs, have been approved (115, 116). Still, rituximab and other anti-CD20 antibodies are being utilized in clinical trials, several of them with the goal of enhancing NK cell cytotoxicity in hematologic malignancies. For example, a recently completed clinical trial tested NK cells, rituximab, plus high-dose chemotherapy, and stem cell transplant for the treatment of recurrent or treatment-resistant B cell NHL (NCT03019640). Allogeneic, expanded cord blood NK cells were administered intravenously five days prior to autologous stem cell transplant, with CD20+ patients receiving rituximab on days 13 to 7 pre-transplant (117). To highlight an “off-the-shelf” approach, the adoptively transferred NK cells were not HLA-matched. 22 patients were enrolled with ages ranging from 15 to 70, a majority of whom were male with diffuse large B-cell lymphoma. Early results reveal an 84% overall response rate (ORR), a 68% relapse-free survival (RFS) rate, and almost 70% of patients in remission 18 months after treatment. No adverse events were associated with the cord blood NK cell infusion, and these cells were shown to exhibit a significantly higher percentage of NKG2D and NKp30 than recipient NK cells. Additionally, the persistence of the transferred NK cells was not affected by HLA mismatch. A similar ongoing trial is combining CD19-targeted, allogeneic CAR NKs with rituximab for the treatment of B cell acute lymphoblastic leukemia (ALL) (NCT05379647).

Like CD20, HER2, a member of the erythroblastic leukemia viral oncogene homologue (ErbB) family, is an antigen with optimal expression patterns for targeting with mAbs. It was originally found to be overexpressed in breast cancer and induce mammary carcinogenesis, but overexpression of HER2 has since been indicated in gastric, esophageal, ovarian, and endometrial cancers (118). HER2 overexpression eventually leads to constitutively activated tyrosine kinases, leading to activation of pathways associated with survival and proliferation, like Ras and PI3K (119). Trastuzumab, a humanized mAb specific to the HER2 extracellular domain (ECD), was first approved for the treatment of HER2+ breast cancer in 1998 (120). In combination with chemotherapy, it led to significant decreases in recurrence and death (both breast cancer and all-cause mortality) and is now being tested in several other HER2-expressing cancers (121). An active phase 1/2a clinical trial for gastric or gastroesophageal junction adenocarcinoma (GJA) is testing a combination of trastuzumab, pembrolizumab, and CYNK-101 cells, though initial results are not yet available (NCT05207722).

Pertuzumab, a more recently developed HER2 humanized recombinant mAb approved for use in various types of breast cancer, blocks a binding pocket required for dimerization, causing inhibition of downstream signalling (122). While its mechanism of action is NK cell-independent, its approval is in combination with trastuzumab. An ongoing clinical trial in the context of breast cancer combines allogeneic NK cells with rituximab and pembrolizumab (NCT05385705). Additionally, two CAR NK cells targeting HER2 are being tested in the context of advanced solid tumors (NCT04319757, NCT05678205), though initial results are not yet available.

EGFR, another member of the ErbB family, is upregulated in glioblastoma multiforme, breast, colorectum (CRC), and lung carcinomas (123). Cetuximab, an anti-EGFR mAb, was approved to treat refractory metastatic CRC in 2004, and late-stage head and neck cancer in 2011. Cetuximab is currently being tested in a phase 1b clinical trial in combination with cytokine-reprogrammed, expanded, cryopreserved, off-the-shelf NK cells termed WU-NK-101 in CRC and squamous cell carcinoma of head and neck (SCCHN) (NCT05674526). When compared with conventional NK cells, WU-NK-101 showed higher expression of activating receptors, Ki67, and Granzyme B (124). In xenograft CRC models, the combination of WU-NK-101 with cetuximab resulted in potent anti-tumor cytotoxicity, as well as increased infiltration and persistence.

A more recent development in the treatment of cancer with mAbs was in the setting of high-risk neuroblastoma, where disialoganglioside GD2 is highly expressed (125). Dinutuximab, or ch14.18 is a chimeric anti-GD2 mAb combining mouse variable genes of the 14.18 mAb with human IgG1 and κ genes (126). Originally, it was tested clinically in the early 2000s and showed no advantage over the conventional therapy (127). However, a retrospective analysis of more than a decade’s worth of follow-up revealed a significant increase in overall survival (128). Largely because of this study, the FDA approved a new standard of care for high-risk neuroblastoma, combining dinutuximab, GM-CSF, IL2, and isotretinoin to treat minimal residual disease (MRD). Recent studies have highlighted further benefit by including the antibody in induction chemotherapy, leading to significant improvements in early responses, tumor volume, and event-free survival (EFS) (129). A phase Ib/II trial is testing another anti-GD2 mAb, naxitamab (approved for use in combination with GM-CSF for neuroblastoma in the bone or bone marrow) in combination with TGFβi NK cells and gemcitabine-based chemotherapy for the treatment of HER2-negative, GD2-positive metastatic breast cancer (NCT06026657) (130). TGFβi NK cells are peripheral blood NK cells that were activated (with IL-2 and K562 cells expressing mbIL21 and 4-1BBL) in the presence of TGFβ, which leads to hyperactive cytokine secretion (131).

Several clinical trials are underway combining activated NK cells with mAbs targeting antigens with more restricted expression across tumor types. For example, daratumumab, a mAb targeting CD38 is being tested in multiple myeloma (MM) in combination with FT576, a B cell maturation antigen (BCMA) specific CAR NK with high-affinity, non-cleavable CD16, and a knockout of CD38 (to prevent fratricide) (NCT05182073). In preclinical models, this dual-targeting approach exhibited superior tumor control compared to treatment with CAR T cells, FT576 alone, or daratumumab alone (132). Early clinical results highlight a lack of dose-limiting toxicities, cytokine release syndrome, neurotoxicity, or GvHD (133). Another ongoing trail approaches fratricide a different way, encouraging it. DR-01, a CD94-specific mAb, is being tested in a phase I/II study in the context of granular lymphocytic leukemia or cytotoxic lymphomas, though results are not yet available (NCT05475925). These cancers of T and NK cells express high levels of CD94, as do healthy T and NK cells. Therefore, through DR-01-mediated fratricide, potent depletion of leukemic cells in preclinical models, and a favorable toxicity profile in non-human primates is observed (134). Despite these approaches to prevent fratricide, it continues to limit therapeutic efficacy of adoptively transferred NK cells, and novel engineering and *ex vivo* activation strategies are needed to overcome it.

Two recent studies in the context of adult T cell leukemia (ATL) utilize mAbs shown to enhance ADCC *in vivo*. A recently completed and published trial utilized alemtuzumab, an anti-CD52 mAb with IL-15, resulting in enhanced NK-mediated ADCC and more durable responses in pre-clinical models (NCT02689453). In humans, the treatment showed no dose-limiting toxicities or severe adverse events, with a 45% ORR and an over 7-fold increase in NK cells ten days post-treatment (135). The other study, an ongoing phase I trial, is attempting to treat ATL with IL-21 expanded, matched donor NK cells and mogamulizumab, an anti-CCR4 mAb (NCT04848064). Preclinical data showed synergy and a two-fold increase in NK-mediated ADCC (136). Mogamulizumab was also tested in combination with IL-15 for ADL and mycosis fungoides/Sezary syndrome in a recently completed phase I trial (NCT04185220). In total, six patients were enrolled, with partial response seen in only one patient and serious adverse events occurring in five (137).

The concept of targeting pan-cancer antigens with mAbs has revolutionized cancer therapy and has opened doors for the optimization of NK cell-mediated antibody-dependent cell cytotoxicity. Tumor-specific mAbs have been pivotal in reducing off-target effects and enhancing the precision of cancer treatment. The pioneering use of rituximab exemplifies the clinical success of mAbs, while the emergence of more recent treatment strategies like dinutuximab and naxitamab for high-risk neuroblastoma highlights the continued evolution of mAb therapies. The synergy between tumor-targeting mAbs and NK cells in the context of cancer offers the potential for more durable responses and better patient outcomes, and this integration continues to be at the forefront of innovative cancer therapies. However, development of resistance to tumor antigen-targeting antibodies as a result of antigen escape is a major barrier to their clinical success. Dual-targeting approaches, such as the combination of daratumumab with an anti-BCMA CAR NK, may prolong or abrogate development of antigen escape, and should be tested further. The subsequent section will delve into the diverse array of checkpoint blockade mAbs, providing further insights into the ever-evolving landscape of NK cell-enhancing strategies.

### 6.2 Checkpoint blockade mAb

A key development in this field of immunotherapy is immune checkpoint blockade (ICB), the use of monoclonal antibodies targeting specific checkpoints to overcome tumor-mediated immune suppression. While many of these mAbs were originally designed with a focus on T-cells, emerging research indicates their potential to influence other crucial components of the immune system, notably natural killer (NK) cells (138, 139). For example, avelumab, an FDA approved mAb targeting PD-L1 for treatment of metastatic Merkel cell carcinoma and urothelial carcinoma, has been shown to rely on T and NK cell-based mechanisms. Avelumab binds PD-L1 on tumor cells or APCs, resulting not only in the blocking of the PD-1 pathway in T cells, but also leading to induction of ADCC by NK cells, similarly to the tumor-targeting mAbs discussed earlier (140, 141). However, ICB has also been shown to enhance NK cell cytotoxicity independent of ADCC.

PD-1 expression is increased on activated NK cells in the TME in several solid and hematologic cancers, where this subset of cells is associated with poor prognosis (142). Upon binding of PD-L1, the activation, cytokine production, proliferation, and cytotoxicity of PD-1+ NK cells are inhibited (40, 143). Following treatment with anti-PD-1 mAbs, the functionality of these NK cells is rescued (144–146). A similar phenomenon is seen with NK cells expressing other immune checkpoints, such as TIGIT, TIM-3, and LAG-3 (42, 147, 148). As such, several ongoing clinical trials are testing the addition of immune checkpoint inhibitors with adoptive transfer of NK cells (NCT05334329, NCT03941262, NCT03388632).

A recent development in the field of NK cell-specific immune checkpoint blockade was the development of monalizumab, a humanized anti-NKG2A blocking antibody. The NKG2A/CD94 heterodimer, expressed on NK and certain CD8+ T cell subsets, binds the non-classical MHC class I molecule HLA-E (149, 150). HLA-E is upregulated in several human cancers and leads to suppression of NK cell antitumor activity when bound by NKG2A/CD94 (151). Monalizumab has been shown to combat this immunosuppression and restore NK cell (and T cell) effector functions (44). As such, monalizumab is undergoing clinical testing in combination with other therapeutics in several tumor settings. For example, a study tested it in combination with cetuximab for treatment of recurrent or metastatic SCCHN. The phase Ib/II study enrolled forty patients who had previously received platinum-based chemotherapy, with a positive toxicity profile (6% grade 3 or 4 adverse events related to treatment) and a 20% ORR (NCT02643550) (152, 153). A phase III study was started based on these results but was discontinued as a result of missing a pre-defined efficacy for threshold (154) (NCT04590963). Another study is testing the combination of monalizumab with trastuzumab for metastatic HER2-positive breast cancer (NCT04307329).

Another ongoing study is testing durvalumab (MEDI4736), an anti-PD-L1 antibody, with monalizumab following chemoradiation for the treatment of stage 3 non-small cell lung cancer (NCT05221840). Early results from this phase III trial, which included 189 patients, show that those who received both mAbs achieved a 35.5% ORR, compared to 17.9% in the group who received durvalumab alone (155). The same treatment strategy is also being tested in extensive stage small cell lung cancer in an ongoing phase II trial (NCT05903092). Another phase II trial is testing monalizumab plus durvalumab in addition to either cetuximab or bevacizumab, an anti-VEGF mAb in several solid tumor settings (NCT02671435). Preliminary results for 18 patients receiving monalizumab, durvalumab, and cetuximab for metastatic microsatellite-stable colorectal cancer reveal a 41.2% ORR, though 100% of patients experienced treatment related AE’s, which were grade 3 or 4 in 77.8% (156).

ICB has emerged as a pivotal development with monoclonal antibodies (mAbs) designed to influence several aspects of the immune system, including NK cells. Clinical trials testing these mAbs in combination with various therapeutics in diverse tumor settings show promise in revolutionizing cancer immunotherapy, highlighting the versatile role of mAbs in enhancing NK cell function.

## 7 Engagers

**Figure 1:**
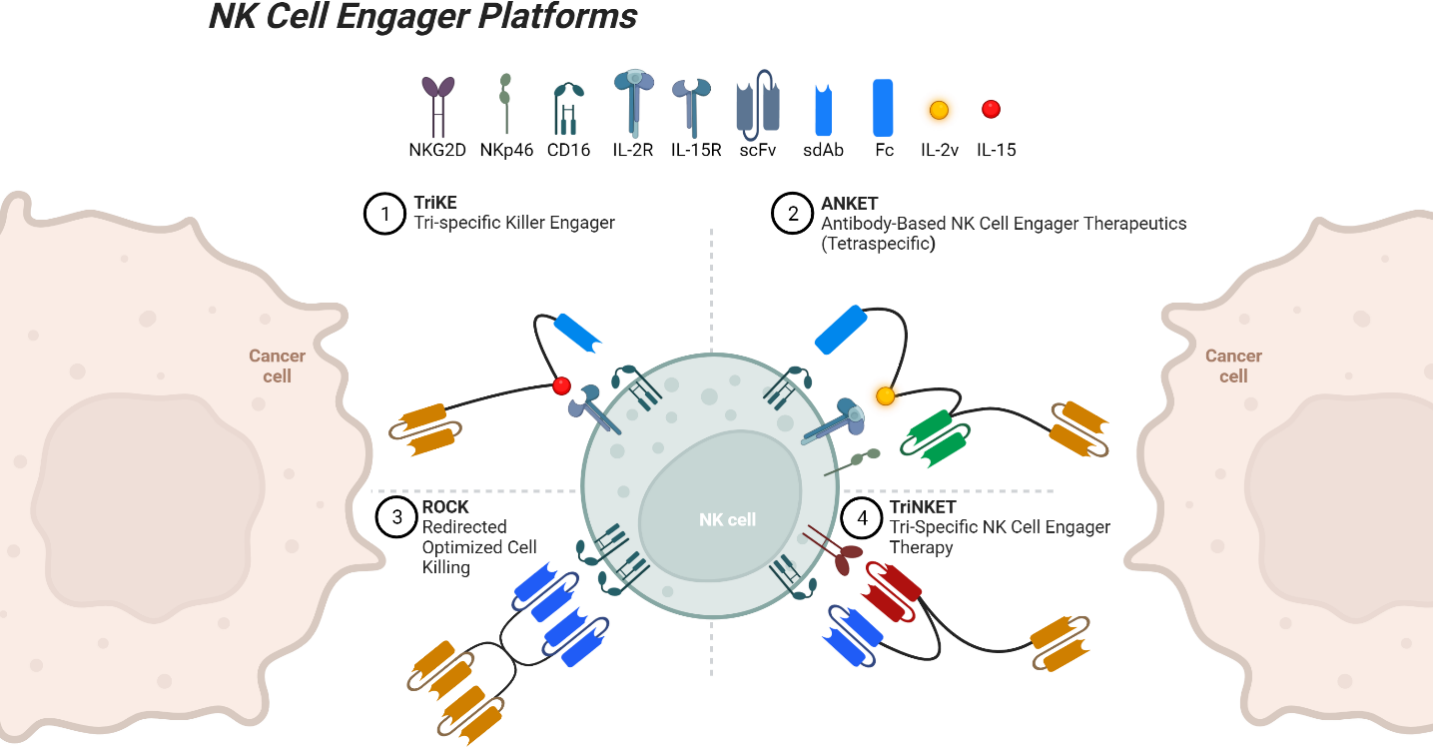
NK cell engager platforms binding both a tumor-associated antigen and an NK cell. Created with Available at: BioRender.com.

NK cell engagers, which include the TriKE, ANKET, TriNKET, and ROCK platforms, among others, are a diverse and promising class of therapeutic agents designed to boost NK cell activity and target malignant cells with precision (see **Figure 1**). These cutting-edge technologies represent a new frontier in the field of cancer immunotherapy, developed much more recently than most of the recombinant cytokines and mAbs discussed earlier. Here, we will discuss each platform, specifically their mechanisms of action, applications in different tumor settings, and the promising results from preclinical and/or clinical studies, shedding light on their role in enhancing the cytotoxic capabilities of NK cells.

Our group has developed an NK cell engager platform is the trispecific killer engager (TriKE), a multi-pronged approach that simultaneously targets tumors, activates NK cells, and facilitates their cytotoxic functions. Second-generation TriKEs contain cam16, a humanized single-domain anti-CD16 nanobody, an IL-15 moiety, and a tumor targeting single-domain antibody (sdAb) or single-chain variable fragment (scFv) (157). TriKEs have been developed and tested that target CD33, CD19, B7H3, EpCAM, mesothelin, and several other tumor-associated antigens (50, 158–161). These molecules improve NK expansion, priming, survival, and cytotoxic activity *in vitro*, *in vivo*, and in early clinical studies. A recent clinical trial tested 161533, a CD33-targeted TriKE, in the setting of myelodysplastic syndromes and AML (NCT03214666). A dose dependent CD16+ NK cell expansion was seen, and four of twelve patients achieved a decrease in CD33+ blast cells (162). This trial tested a first-generation TriKE, which contained an anti-CD16 scFv, but was terminated due to development and improved functionality of the second-generation TriKE (163).

Camelids, such as camels, llamas, and alpacas, as well as cartilaginous fish like sharks, possess immunoglobulins that consist exclusively of heavy chains (164). Their smaller size equips them with superior tissue-penetrating capabilities, and their variable length CDR3 regions allow for binding in deeper grooves when compared to conventional antibodies, prompting interest in their use to treat human diseases (165). The TriKE platform stands out for its utilization of a camelid sdAb/nanobody, resulting in enhanced IL-15 potency when compared to first-generation TriKE, which included a CD16 scFv (157, 163). To date, caplacizumab, a bivalent nanobody designed to target von Willebrand factor for treating thrombotic thrombocytopenic purpura and thrombosis, remains the only FDA-approved nanobody (166). However, a CAR T cell armed with two nanobodies that target BCMA is approved for the treatment of multiple myeloma, and an anti-PDL1 nanobody has gained approval in China.

Another NK engager platform, the tetraspecific ANKET (antibody-based NK cell engager therapeutics), contains an IL-2 variant that inhibits Treg stimulation, an antibody domain to a tumor antigen, an antibody domain to NKp46, and the Fc domain of IgG1 that binds CD16 (54). A CD20-targeting ANKET termed IPH6501 showed increases in NK cell proliferation, cytotoxicity, and chemokine and cytokine secretion (167). It will soon be tested in a phase I/II clinical trial for patients with NHL (NCT06088654). A trispecific ANKET, SAR445514, targeting BCMA without an IL-2 domain, is currently being tested against relapsed MM and refractory light-chain amyloidosis (NCT05839626). Another trispecific ANKET, SAR443579, targeting CD123, is also currently being tested in a phase I/II trial in various hematologic malignancies (NCT05086315). Three TriNKET (trispecific NK cell engager therapy) molecules are also undergoing clinical testing, targeting HER-2, CD33, and BCMA, though little information is publicly available for these molecules (NCT04143711, NCT04789655, NCT04975399).

The final platform, ROCK (redirected optimized cell killing) consists of tetravalent bi-specific engagers. A recently completed phase II clinical trial testing AFM13, a ROCK molecule targeting CD16 and CD30, treated 25 patients with relapsed or refractory classical Hodgkin lymphoma (NCT02321592). Only two patients suffered treatment-associated serious adverse events, which were both completely resolved. The ORR was 16.7%, and 12-month PFS was seen in 12.6% of patients (168). AMF13 is also being tested in a phase II trial for mycosis fungoides, with preliminary results showing a 24.1% ORR (NCT04101331) (169). A third, phase I/II trial is testing AFM13 in combination with cord blood-derived NK cells for the treatment of CD30+ Hodgkin and NHL (NCT04074746). Preliminary results are very promising, with a 100% ORR, and 62% complete response rate after 2 cycles of the recommended phase 2 dose (170). Strikingly, the enrolled patients had a median of seven prior forms of treatment. Of note, as all engagers discussed in this section rely on activation through CD16, ADAM17-mediated cleave remains a barrier to therapeutic efficiency and may benefit from the addition of an ADAM17 inhibitor.

## 8 Synergistic combinations with NK cell products

The therapeutic strategies discussed here, including cytokines, monoclonal antibodies, and NK cell engagers, possess the ability to stimulate endogenous NK cell populations within a tumor. Ideally, this intrinsic mechanism would be sufficient to drive positive clinical outcomes. However, practical challenges arise as the development of tumor-resistance to endogenous NK cells and widespread immunosuppression hampers the effectiveness of therapeutics aimed to stimulate NK cells *in situ* (171). Therefore, these strategies can benefit from adoptive transfer of NK cells, introducing two crucial considerations: first, ensuring that the administered NK cells effectively migrate to the tumor, and second, ensuring their sustained persistence for optimal therapeutic impact.

A recent focus in the field of NK cell therapy has been to expand NK cells in a way that allows for optimal persistence and infiltration. As such, several forms of NK cell products exist that vary in their source, activation, and function, several of which were referenced earlier in this review, in combination with therapies aimed to enhance cytotoxicity. Therapeutic NK cell products can be derived from placental/umbilical cord blood, peripheral blood, induction of stem cells, or immortalized cell lines, which then can be expanded using a variety of methods. For example, NK cells can be expanded *in vivo* and stimulated with IL-2, generating cytokine-induced memory-like NK cells that have been shown to persist in patients for over six months (172, 173). Alternatively, NK cells can be expanded using feeder cells, like K562 expressing membrane-bound IL-21 and 4-1BB ligand, which interact with NK cell-surface receptors to induce proliferation and activation (174, 175). Alternative genetic alterations to the K562 feeders can further change characteristics of the NK cells, such as the increased persistence seen when K562 cells contain membrane bound IL15 and 4-1BB ligand (176). Additionally, the expression of CCR7 on K562s enhances homing of NK cells to lymph nodes via CCR7 trogocytosis in preclinical models (177). Epstein-Barr virus-transformed lymphoblastoid cell lines have also been used to successfully expand human NK cells (178). NK cell products can also be expanded without feeder cells, such as those generated following enrichment of placental stem cells and subsequent culture with cytokines including IL-15 (179).

When integrating a therapeutic strategy aimed at boosting NK cell cytotoxicity with the adoptive transfer of NK cells, the selection of an appropriate expansion process is crucial to generate NK cells that align synergistically with the therapeutic approach. For example, in the context of monoclonal antibodies and TriKEs, which exert their effects through cross-linking of CD16, the expansion process must retain expression of CD16 on the NK cells destined for administration with these therapies. In this context, NK-92 cells (an immortalized NK cell line used in some clinical trials), which have no CD16 expression, and stem cell-derived NK cells, which have low endogenous CD16 expression, would not be logical choices to combine with monoclonal antibodies or TriKEs (180). However, genetic engineering could alleviate this issue.

Another important issue to consider when selecting an NK cell product is the use of autologous or allogeneic NK cells. In general, adoptive transfer of allogeneic NK cells may outperform autologous ones for several reasons. First, autologous NK cell activity is downregulated due to expression of inhibitory KIR and NKG2A, which bind self-HLA present on tumor cells and may exhibit functional deficits from widespread immunosuppression (181). Second, autologous cell therapies are notably more labor- and time-intensive due to the requirement that they are generated individually for each patient, while allogeneic NK cells could be generated for “off the shelf” use (182). However, autologous NK cell infusions offer certain advantages, as they may eliminate the requirement for lymphodepletion, mitigate fratricide, and avoid potential challenges associated with HLA-mismatch (183).

In conclusion, while therapeutic strategies aiming to stimulate endogenous NK cell populations show promise, practical challenges such as tumor resistance and immunosuppression necessitate the consideration of adoptive transfer of NK cells. The efficacy of these strategies relies on the careful selection of suitable expansion processes, considering factors such as the consistent maintenance of surface receptor expression that complements the therapeutic strategy, the necessity for genetic engineering, and the decision between utilizing autologous or allogeneic NK cells. Achieving optimal persistence, infiltration, and compatibility with specific therapeutic approaches is essential for maximizing the therapeutic impact of NK cell-based strategies in cancer treatment.

## 9 Conclusion

In this comprehensive review, we have delved into the landscape of therapeutic approaches aimed at enhancing NK cell cytotoxicity, encompassing fundamental insights into NK cell biology, immunosuppression within the TME, and the arsenal of innovative strategies for fortifying NK cell activity. However, toxicity and off-target effects remain barriers that need to be overcome. The development of molecules with refined binding specificities, like IL-2 that doesn’t interact with Tregs, and alternative methods of administration, such as through oncolytic adenoviruses, allows for greater precision and safety. More research is needed in this area to further the clinical translation of NK-based therapies.

Our exploration has shed light on the potential of these strategies to revolutionize cancer immunotherapy, from the foundational understanding of NK cell biology to the clinical translation of advanced therapies. The advent of cytokines, monoclonal antibodies, and NK cell engagers has offered a diverse toolbox to empower NK cells and bolster their cytotoxicity. Investigation into these therapeutic approaches leaves us with a sense of optimism and confidence in the future of NK cell-based immunotherapy. We are hopeful in the potential to improve patient outcomes and reshape the landscape of oncology.

## Author contributions

TS: Writing – original draft, Writing – review & editing. JM: Writing – review & editing.
